# An Innovative Lab-Based Training Program to Help Patient Groups Understand Their Disease and the Research Process

**DOI:** 10.1371/journal.pbio.1002067

**Published:** 2015-02-10

**Authors:** Marion Mathieu, Constance Hammond, David G. Karlin

**Affiliations:** 1 Tous Chercheurs, Marseille, France; 2 Institut de Neurobiologie de la Méditerranée, INMED UMR 901 Inserm, Marseille, France; 3 Aix-Marseille Université, Marseille, France; King's College London, UNITED KINGDOM

## Abstract

Genuine partnership between patient groups and medical experts is important but challenging. Our training program meets this challenge by organizing hands-on, lab-based training sessions for members of patient groups. These sessions allow “trainees” to better understand their disease and the biomedical research process, and strengthen links between patients and local researchers. Over the past decade, we and our partner institutes have received more than 900 French patients, with the participation of over 60 researchers and clinicians.

## Abstract 2

Lab-based mini-research projects allow members of patient groups to understand the scientific method and how research works in practice, thereby fostering genuine partnership between patient groups and medical experts.

This Community Page is part of the "Public Engagement in Science" Series.

## Introduction

Over the past twenty years, patient groups have become partners of biomedical research, which they support and often fund [1–5]. They help researchers by bringing a source of expertise grounded in real-life experience [6,7]. For that reason, they are sometimes called “patient-experts” [8]. Patient groups also facilitate recruitment for clinical trials, help to catalyse collaborations, and disseminate research results [9]. Their influence can be invaluable in overcoming regulatory or political obstacles [10]. Good relationships between patient groups and researchers can prevent or attenuate controversies that regularly occur regarding research results or practices [11,12,13]. Last but not least, working with them can be hugely rewarding and inspiring [14,15].

However, genuine dialogue between patient groups and medical experts is difficult for a wide range of reasons. Persons afflicted with diseases, though they are expert of their clinical symptoms, often lack the biological knowledge necessary for understanding the basic mechanisms underlying their disease and the corresponding research questions [10]. Even when they have been taught biology, their knowledge may be outdated, given the very fast pace of scientific advances [16]. In addition, patients often have a poor understanding of scientific methods (in particular the experimental approach) and of how scientific results are constantly challenged (see Table 1 for a list of frequent misconceptions). An additional concern comes from the research process itself, which generates a pressure for researchers to “hype” their own research [17,18]. These issues can lead either to a general mistrust of research, or to an exaggerated trust in nonvalidated therapies. Finally, patient groups who fund research have a particularly crucial need to understand how it works in practice, since they need to have informed discussions with their scientific board (composed of researchers and clinicians) to allocate their funds wisely.

**Table 1:** Aspects of the scientific method that patients often understand poorly.

Aspects poorly understood	Comments	Notions explained during the training
The time scale of research	The time scale of research is always too long when compared to the life expectancy of a sick child or adult.	Patients experience by themselves the duration of experiments, the necessity of carrying out control experiments, and replicates.
The provisional nature of scientific knowledge	Members of patient groups can get unrealistic expectations about a hyped research avenue.	We give examples of promising or attractive research avenues that did not work out and emphasize the need to replicate published research.
The interconnection of research fields	Members of patient groups often do not realize that allocating funds to research on a given disease may benefit other diseases.	We give examples related to their disease. For instance, in a session on dystonia, we explained how deep brain stimulation, designed at first for essential tremor, is now used for dystonia and many other neurological diseases [19].
The importance of animal models	Members of patient groups know little about animal models used in labs and often think that apes are the only relevant models for human diseases.	We present various animal models available for their pathology and how researchers choose models. We experiment either on a model organism of their disease or on the simplest genetically modified organism, bacteria. We present the difficulties, limits (practical, legal, and ethical), and costs of animal models.
The cost of research	Members of patient groups often have a poor idea of research costs, despite the fact that they often fund research.	We give examples of the cost of equipment and consumables they use (microscopes, enzymes...), and of building and running a lab.

Several initiatives have been set up to answer these needs, such as the Eurordis Summer School and e-learning courses (www.eurordis.org/training-resources), the European Patients' Academy on Therapeutic Innovation (EUPATI, www.patientsacademy.eu/index.php/en), and the Genetic Alliance programmes (www.geneticalliance.org/programs). These programs aim at improving medical literacy [20], at enabling patients to engage with data and information that are relevant to them, at connecting them with others who may have relevant experience or expertise, or at facilitating mutual support. The EUPATI program also provides training courses to help patients become effective advocates and advisors in the treatment development process. These training sessions consist in e-learning and face-to-face courses, including case studies.

We present herein a complementary training program based on an immersion in a research lab. Indeed, an important aspect of research, the experimental method, can only truly be understood by performing experiments, ideally over several days [21]. For that reason, we set up lab-based training sessions in biology and research practices in the form of 3-day mini research projects. Over the past ten years, these have been attended by more than 900 French patients, with the help of over 60 researchers and clinicians, and have been implemented in several public engagement institutes and research centres. We outline below the target audience, the aims of the sessions, and their design. Finally, to help researchers who wish to set up a similar initiative, we present elements that the trainees and participating researchers find particularly beneficial, on the basis of evaluations.

## The Trainees

Tous Chercheurs, a French association founded by biomedical researchers in Marseille [21], created this training programme for patient groups in May 2004. It originated in a dedicated lab inside a biomedical research institute, the Mediterranean Institute of Neurobiology (INMED). Since 2007, we have piloted a national network that includes four other training centres (the DNA schools of Evry-Généthon, Angers, Nîmes, Poitiers), which all use the same format for the training program. This network allows us to share skills and resources: for instance, the members of the network regularly receive patient groups concerned with different diseases and exchange information and advice on how to adapt training sessions for a particular disease. All trainers have at least an MSc (most have a PhD) and two years’ research experience, and are public engagement professionals.

Whenever possible, the training centres call on local researchers to participate in the training session as experts specialised in the diseases considered, thereby strengthening the links between patients and local research institutes or medical departments.

## Target Audience

Training sessions are open to patient groups suffering from chronic diseases (including rare genetic diseases, familial cancers, autoimmune, and inflammatory diseases). We offer training to organisations rather than to individuals because this makes it easier to set up a group of patients concerned by the same disease, and to advertise the sessions (excellent advice on how to contact patient groups can be found in a recent article [16]). Sessions are open to adults and to teenagers ages 14 and up (accompanied by their parents) and are, of course, accessible to the physically disabled. No prior knowledge of biology is required. The training sessions have between 8 and 12 trainees.

## Main Aims of the Training Sessions

Our main goals are to allow patients to better understand the research process (including its limitations) and to strengthen links between patients and local researchers or clinicians, so that patient groups can contribute to the research enterprise in the most fruitful way possible.

Our sessions are designed so that patients can:

- learn some of the basic principles of biology, by means of hands-on experiments;
- receive a clear explanation as to the origin and mechanisms of their disease;
- understand the scientific method, but also how research works in practice;
- understand the limitations of research studies and learn to have a critical eye on research results;
- ask all the questions they do not have time to ask during a medical consultation;
- and discuss new avenues for research and possible treatments with an expert, in a friendly setting.

## Overview of the Training Sessions

We offer two types of training sessions: one that addresses genetic diseases, and one for autoimmune or inflammatory diseases. They each follow a similar format, which is comprised of three parts: 1) an introductory session (one half day), composed of a case study and of a short biology primer; 2) a set of practical workshops (two days); and 3) a roundtable with an expert (one half day). During the session, we also organize a social event (e.g., a picnic by the sea), which gives trainees a good opportunity to reflect together about what they have learned and to discuss follow-up activities.

We use an inquiry-based approach [22,23], similar to the one that we designed for high school students [21]. Trainees are active participants of a mini research project. Over the course of the workshops, they learn how to identify research problems, emit hypotheses, test them, and interpret and discuss their results, as researchers would do. All sessions start with a case study in the form of biomedical documents such as familial trees or results of medical tests. Trainees are encouraged to analyse them in order to formulate hypotheses and to define possible research questions, some of which will be tested during the practical workshops. We provide them with the theoretical knowledge that they need as the course proceeds.

We also discuss the pressure for researchers to “hype” their own research, including in the primary scientific literature, and provide memorable evidence of the provisional nature of scientific results. For instance, we show them two articles published a year apart in a prestigious journal, with titles reporting completely opposed conclusions.

We work in close partnership with the patient group(s) and the clinicians and researchers specializing in that disease. Prior to the training session, we ask them what questions they would like us to clarify (see Table 2 for common questions). We then design the practical workshops depending on these requests, but also to reflect current research on their disease. We will take the example of the genetic disorder Primary Ciliary Dyskinesia (PCD) to illustrate our approach.

**Table 2:** Examples of common questions that trainees and experts would like us to clarify during the training session.

Frequent questions of trainees in relation with genetic diseases
- How can one identify a gene(s) associated with a disease?
- What is the relationship between mutations and symptoms?
- How do mutations cause a disease?
- Why does the diagnosis take so long?
- Why are results of research involving my biological samples not systematically communicated to me?
- Is there any research on my disease?
- Now that the gene has been discovered, why is there no treatment?
- What is the origin of my mutation (why and how did it happen?)
- How is the mutation transmitted between generations?
**Frequent questions of trainees in relation with autoimmune and inflammatory diseases**
- Can I transmit my autoimmune disease to my children?
- I do not have a given disease predisposition gene marker, yet I have the corresponding disease—is that possible? How come? (For instance, I am not HLA-B27 but I am told I have spondylarthritis. Is it possible? Why?)
- What proportion do genes play in my disease?
- What are good biological markers of my disease?
- How can I predict outbreaks of my disease?
- How do biotherapies work?
**Frequent issues faced by researchers in communicating with patients about research, diagnosis, and treatments**
Patients often do not understand:
- the biological causes of a disease (and thus what can be done to remediate it);
- that researchers cannot answer all their questions;
- how clinical trials work in practice;
- the time scale of research, which is not the same for patients and for researchers;
- that to be included in a long-term trial implies not only to not be included in other trials during that time but also during a certain period after the end of the trial (period of “noninclusion”); the necessity of placebos.

## Training Sessions for Patient Groups with Genetic Diseases: Example of Primary Ciliary Dyskinesia (PCD)

In PCD, the structure and/or function of motile cilia is altered, leading to a buildup of mucus in the lungs and to chronic respiratory infections, as well as a variety of other issues including loss of hearing and infertility [24]. Over twenty genes associated with PCD, encoding cilia proteins, have been discovered, but many remain to be identified [25]. At present, a diagnosis is reached by observing cilia by microscopy. Trainees articulated the following questions: how to identify genes implicated in PCD; whether a genetic diagnosis can be made; what is the relationship between mutations and the symptoms of the disease; what is the function of both normal and diseased cilia; and what research is done to develop treatments or improve disease management. In addition, the participating specialist asked us to clarify why identifying genes implicated in PCD is slow and complex, as well as the principle and limits of molecular diagnosis of PCD, should it become feasible.

**Fig 1:**
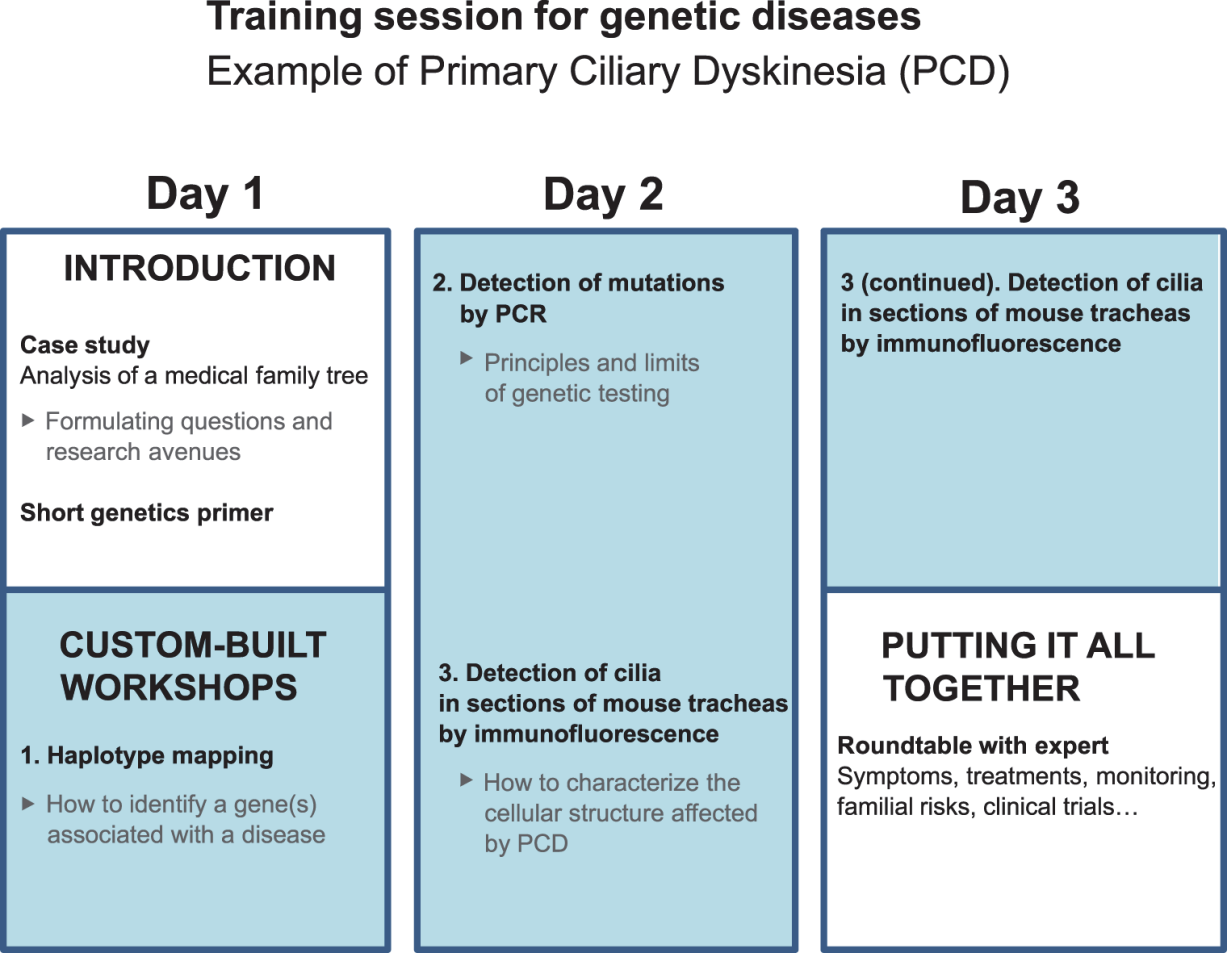
Layout of a typical training session for patient groups with a genetic disease. See also Fig. 2 for how we tailor each training session to a particular disease.

**Fig 2:**
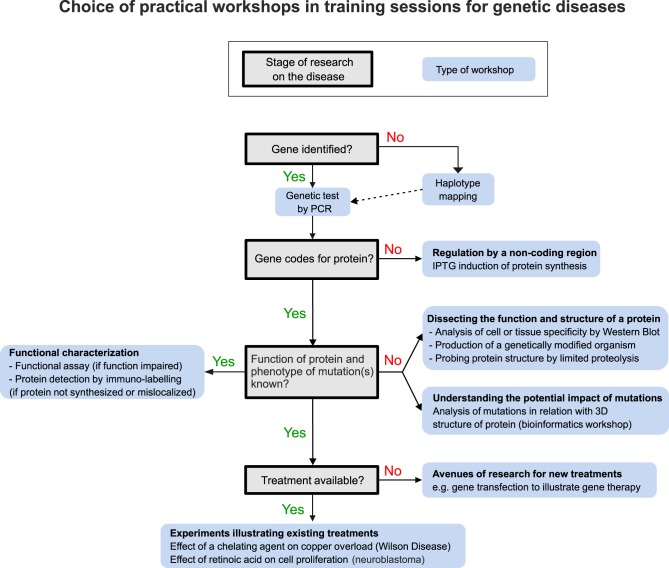
How we tailor practical workshops for a given disease based on the stage of research.

Fig. 1 summarizes the training session that we organized for PCD, which comprised a case study, three practical workshops, and a roundtable with an expert. Fig. 2 illustrates how we tailored the practical workshops to reflect current research. Since many genes associated with PCD have not yet been identified, we organized a haplotype mapping workshop, followed by a PCR (polymerase chain reaction)-based workshop to demonstrate genetic testing (Fig. 1). To illustrate the function and pathogenesis of cilia, we designed a workshop centred on visualizing the cilia and one of their constituent proteins (Fig. 1).

### Case Study

The case study, which was centred on the observation of a familial tree (Fig. 1), served as a starting point for a discussion around genetics and introduced many of the notions addressed during the rest of the session. In addition, this initial period of reflection allowed us to dispel any prejudice that trainees might hold regarding their disease. After the case study, we provided trainees with basic principles of genetics by means of simple experiments, such as visualizing cells and chromosomes and extracting their own DNA.

### Haplotype Mapping

The haplotype mapping workshop simulated the identification of a genomic region bearing a candidate gene. Trainees much appreciated the fact that it is this very approach that allowed the identification of the two most frequent mutated genes identified in PCD, DNAH5 and DNAI1 [26,27].

### Simulation of Genetic Testing by PCR

Many of the trainees’ questions prior to the training session revolved around genetic tests (even though at present the diagnosis is made by microscopy). In response to this, we organized a workshop comprising the detection of a length polymorphism by PCR in order to clarify the principles and limits of genetic tests. This workshop also formed a forum for explaining the principle of gene therapy (a subject of research in PCD).

### Visualization of Cilia by Immunofluorescence

The last workshop was structured around visualization of cilia by immunofluorescent detection of one of their constituent proteins (Fig. 1). It helped to clarify what proteins are, a subject often poorly understood by trainees, and the link between protein expression and physiology. It also prepared the trainees for the discussion with the expert on the impact of mutations on the function and structure of cilia.

### Roundtable

Finally, during the roundtable (Fig. 1), trainees asked questions about the latest news from research on their disease, covering many aspects: the identification of new causative genes; progress in understanding of the relation between genotype and phenotype, including infertility, hearing loss, and neurological problems; and new strategies for diagnosis and treatment (gene therapy). The expert also presented and discussed some of the issues he encountered in his research—such as difficulties in meeting ethical requirements for gene therapy trials on model mice.

## Training Sessions for Patient Groups with Autoimmune Diseases

**Fig 3:**
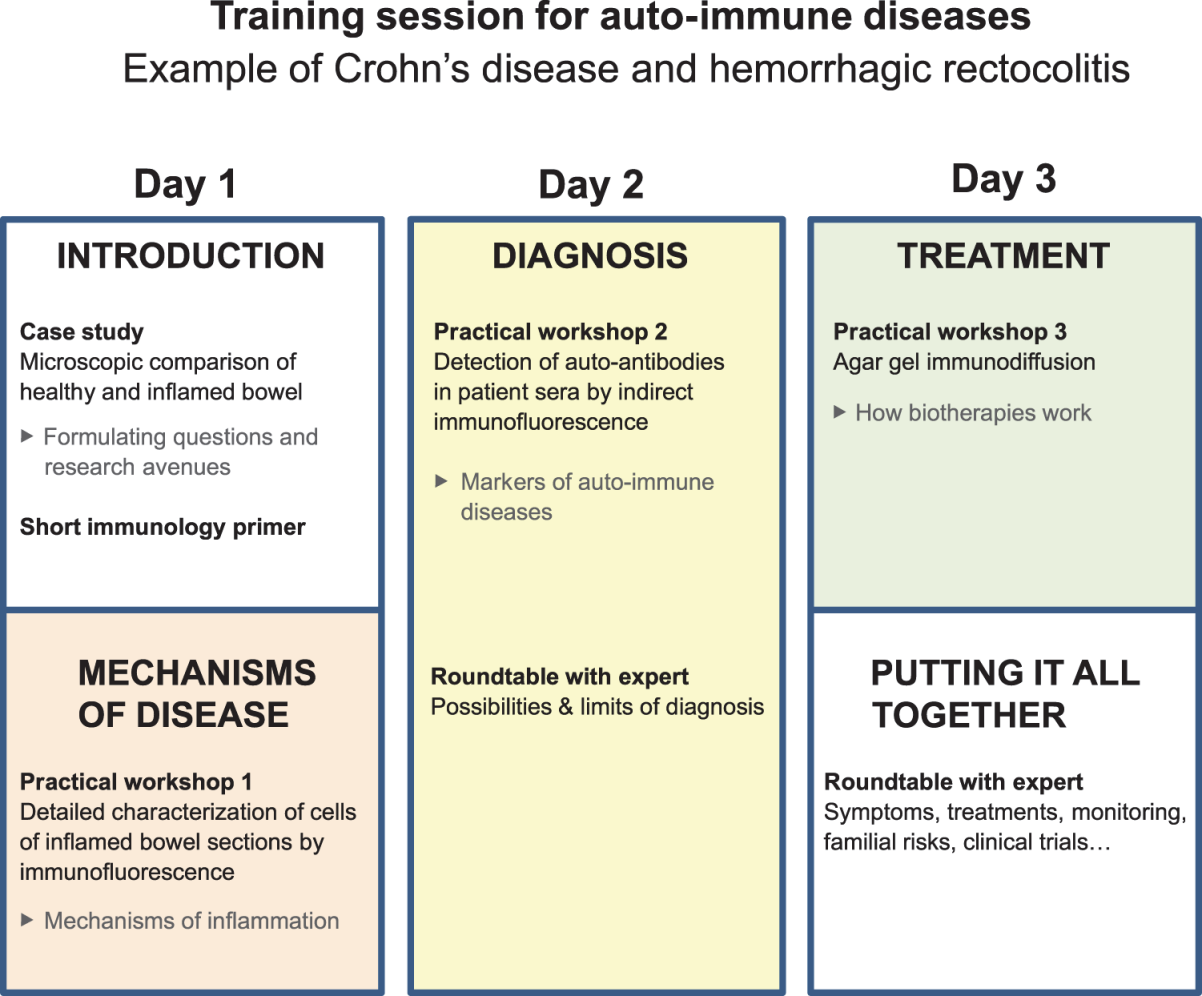
Layout of a typical training session for patient groups with an autoimmune or autoinflammatory disease.

Fig. 3 describes a training session for autoimmune and inflammatory diseases developed for patient groups concerned with Crohn’s disease, hemorrhagic rectocolitis, and celiac disease. The general layout of the session is similar to that of genetic diseases (Fig. 1). However, the expectations of trainees (Table 2) tend to be much more uniform than in genetic disease sessions. Consequently, the training sessions for autoimmune and inflammatory diseases are always comparable to that presented in Fig. 3, with minor adaptations.

## Funding

Patient groups are not charged for their participation in the workshops. However, each session costs about €3,200 to run. The cost includes the salaries of trainers (about €2,500, which comprises the design of the session, the preparation of the experiments and the actual teaching), consumables (typically around €500), and expense of invited experts if they come from other cities. The cost per trainee is clearly higher than that of the other training programs mentioned in the introduction, owing to the experimental nature of the sessions. Over the years we have obtained funding from a wide variety of sources, including charities, research-funding bodies, and local councils (listed on http://touschercheurs.fr). Our funders appreciated the fact that we filled an important, but hitherto unmet, need. They also appreciated the fact that our training programme has achieved national recognition, and that we have provided researchers with valuable experience in public engagement.

## Evaluation

We evaluate each session on the basis of qualitative and quantitative feedback provided both by trainees and by participating experts. Quantitative indicators are in S1 Table. Trainees expressed very high satisfaction with the programme (18.5/20); details of individual questions are in S2 Table. Another sign of satisfaction was that we organized repeat sessions for a third of the patient groups that participated in the sessions. Trainees particularly appreciate the hands-on nature of the sessions, which allows them to better understand theoretical concepts and how research works in practice (S1 File presents representative comments). They also appreciate being able to enter into a genuine dialogue with a medical expert in a relaxed environment and making their opinions heard.

As for the specialists who participate to the sessions, they find the interaction with patients to be a strong source of motivation for their research, in particular by seeing applications of their studies in a very concrete way (see comments in S1 File). For instance, one expert reported “I had never become aware of the war that families (of patients afflicted with cystic fibrosis) wage against the opportunistic bacterium *Pseudomonas aeruginosa*. I left more convinced than ever of the usefulness and necessity of finding alternatives to antibiotics to fight this pathogen. This is very rewarding for our team, despite the fundamental nature of our work.” (S1 File). Researchers can also witness how much patients appreciate hearing an expert say “I don’t know.” Several specialists who are members of the scientific board of a patient group mentioned that they had discussions of greater quality with trainees who had attended the session, owing to their improved understanding of the research process and of the biology of their disease.

Other evidence that the programme is successful (S3 Table) includes a national award, a request from the French National Institute for Medical Research (Inserm) to organize further training programmes for patient groups, and the demand for a transfer of skills from several organizations.

## Recommendations for a Successful Training Session

On the basis of evaluation and of our experience, we recommend that sessions:

- are tailored to the specific condition(s) of trainees, by consulting them ahead of the session;
- take place over several days, as this gives trainees enough time to conduct their own experiments and to assimilate new information. It also gives them a realistic idea of the time scale of research (which is always much longer than they imagined!). In addition, this time frame gives them the opportunity to come up with new questions to the expert, based on their newly acquired knowledge;
- take place on or near a scientific campus. This makes it easy to attract academic experts of the diseases considered and to have access to technical support. It also allows the trainees to discover how a research lab and scientific campus are organized.

## Supporting Information

S1 Table Evaluation indicators for the training sessions. Details of individual questions are in S2 Table.
(DOC)

S2 Table Evaluation form for training sessions on genetic diseases. The form presented is the standard one for sessions on genetic diseases. A minor variation between sessions is that there can be one or two experts participating to the sessions (for instance a physiotherapist in the session for PCD). The notes given are the average of the years 2012–2013, for the training sessions organised by Tous Chercheurs, corresponding to the first column of S1 Table.
(DOC)

S3 Table Other indicators of success of the training programme. (DOC)

S1 File Testimonies from trainees and researchers (translated from French). (DOC)

## Abbreviations

EUPATI: European Patients' Academy on Therapeutic Innovation
PCD: Primary Ciliary Dyskinesia
INMED: Mediterranean Institute of Neurobiology
PCR: polymerase chain reaction
