# Supplementary material for: An Innovative Lab-Based Training Program to Help Patient Groups Understand Their Disease and the Research Process
Source: PLoS Biol. 2015 Feb 10;13(2):e1002067. doi: 10.1371/journal.pbio.1002067 (PMC4323103; doi:10.1371/journal.pbio.1002067)
Supplement: S1 Table — Details of individual questions are in S2 Table. (DOC) [file pbio.1002067.s001.doc]

**Supporting Table S1: Evaluation indicators for the training sessions**

Details of individual questions are in Supporting Table S2.

| **Number of:** | **Tous Chercheurs** | **Other partners^(1)^** | **Total** |
| --- | --- | --- | --- |
| Training sessions | 61 | 40 | 101 |
| Proportion of repeat sessions: | 20/61 (34%) | 13/40 (33%) | 33/101 (33%) |
| Trainees | ~500 | ~400 | ~900 |
| Global satisfaction from trainees ^(1)^ | 18.5/20 ^(2)^ | 18.7/20 ^(3)^ | 18.6/20 |

^(1)^ DNA schools of Angers, Evry-Génethon, Nîmes, Poitiers.

^(2)^ years 2012 and 2013; mean of the last 13 latest training sessions, on 105 trainees.

^(3)^ years 2012 and 2013; mean of the last 6 latest training sessions, on 50 trainees.
