# Supplementary material for: An Innovative Lab-Based Training Program to Help Patient Groups Understand Their Disease and the Research Process
Source: PLoS Biol. 2015 Feb 10;13(2):e1002067. doi: 10.1371/journal.pbio.1002067 (PMC4323103; doi:10.1371/journal.pbio.1002067)
Supplement: S2 Table — The form presented is the standard one for sessions on genetic diseases. A minor variation between sessions is that there can be one or two experts participating to the sessions (for instance a physiotherapist in the session for PCD). The notes given are the average of the years 2012–2013, for the training sessions organised by Tous Chercheurs, corresponding to the first column of S1 Table. (DOC) [file pbio.1002067.s002.doc]

**Supporting Table S2: evaluation form for training sessions on genetic diseases**

The form presented is the standard one for sessions on genetic diseases. A minor variation between sessions is that there can be one or two experts participating to the sessions (for instance a physiotherapist in the session for Primary Cilia Dyskynesia (PCD)).

The notes given are the average of the years 2012-2013, for the training sessions organised by Tous Chercheurs, corresponding to the first column of Supporting Table S1.

Tick the circle Ο for the reply closest to your opinion.

| **What global note would you give to the training session ? .... / 20** | | | | **18.5/20** |
| --- | --- | --- | --- | --- |
|  |  | **1 2 3 4 5** |  |  |
| How would you rate the level of difficulty | Too simple | **Ο Ο Ο Ο Ο** | Too difficult | 3.1/5 |
| Did the trainers adapt to your level? | Very little | **Ο Ο Ο Ο Ο** | A lot | 4.8/5 |
| Are you satisfied with the balance between theory and practice? | Not at all | **Ο Ο Ο Ο Ο** | Completely | 4.8/5 |

| **Content** | | | |  |
| --- | --- | --- | --- | --- |
| Did the training session clarified the following concepts : | | | |  |
| Cells | Not at all | **Ο Ο Ο Ο Ο** | Completely | 4.5/5 |
| Chromosomes, DNA | Not at all | **Ο Ο Ο Ο Ο** | Completely | 4.5/5 |
| Gene | Not at all | **Ο Ο Ο Ο Ο** | Completely | 4.4/5 |
| Mutation | Not at all | **Ο Ο Ο Ο Ο** | Completely | 4.4/5 |
| Protein | Not at all | **Ο Ο Ο Ο Ο** | Completely | 4.2/5 |
| Genetic Tests | Not at all | **Ο Ο Ο Ο Ο** | Completely | 4.5/5 |
| The timescale of research | Not at all | **Ο Ο Ο Ο Ο** | Completely | 4.4/5 |
|  |  |  |  |  |
| Are there concepts that remain unclear after the training session? | Very few | **Ο Ο Ο Ο Ο** | A lot | 1.7/5 |
| If yes, which ones? ………………………………………………………………………………………………….  …………………………………………………………………………………………………………………………  ………………………………………………………………………………………………………………………… | | | |  |
| Are there additional notions that you would have liked to see covered during the training sessions? | Very few | **Ο Ο Ο Ο Ο** | A lot | 1.6/ |
| If yes, which ones? ………………………………………………………………………………………………….  …………………………………………………………………………………………………………………………  ………………………………………………………………………………………………………………………… | | | |  |

**The trainers**

| Globally, did the trainers explain clearly? | Not at all | **1 2 3 4 5**  **Ο Ο Ο Ο Ο** | Very well | 4.9/5 |
| --- | --- | --- | --- | --- |
| Did they organise the workshop interactively? | Not at all | **Ο Ο Ο Ο Ο** | Very well | 4.9/5 |
| Did the trainers reply well to your questions? | Not at all | **Ο Ο Ο Ο Ο** | Very well | 4.7/5 |
| Are there points on which the trainers could improve? | Yes | **Ο Ο** | No | - |

If yes, which ones ?

……………………………………………………………………………………………………………………….………………….…

………………………………………………………………………………………………………………………………………….…

| **The physiotherapist** | | | |  |
| --- | --- | --- | --- | --- |
| Globally, are you satisfied with his intervention? | Not at all | **Ο Ο Ο Ο Ο** | Completely | 4.9/5 |
| Did you find it useful? | Not at all | **Ο Ο Ο Ο Ο** | Completely | 4.8/5 |
| Are you satisfied with the balance between theory and practice? | Not at all | **Ο Ο Ο Ο Ο** | Completely | 4.7/5 |
| Are there topics that you could not bring up with him? | Yes | **Ο Ο** | No | - |
| If yes, which ones?  ……………………………………………………………………………………………………………………….  ……………………………………………………………………………………………………………………….  ………………………………………………………………………………………………………………………. | | | |  |
| **The expert** | | | |  |
| Globally, are you satisfied with his intervention? | Not at all | **Ο Ο Ο Ο Ο** | Completely | 4.6/5 |
| Did you find it useful? | Not at all | **Ο Ο Ο Ο Ο** | Completely | 4.6/5 |
| Are there topics that you could not bring up with him? | Yes | **Ο Ο** | No | 4.7/5 |
| If yes, which ones?  ……………………………………………………………………………………………………………………….  ……………………………………………………………………………………………………………………….  ………………………………………………………………………………………………………………………. | | | |  |
| **Do you have suggestions to improve the training session?**  ……………………………………………………………………………………………………………………….  ……………………………………………………………………………………………………………………….  ……………………………………………………………………………………………………………………….  ……………………………………………………………………………………………………………………….  ………………………………………………………………………………………………………………………. | | | |  |

| **"Post-session "** |  | **1 2 3 4 5** |  |  |
| --- | --- | --- | --- | --- |
| Did this training session allow you to acquire the bases of biology you felt you lacked? | Not at all | **Ο Ο Ο Ο Ο** | Completely | 4.6/5 |
| Do you think this training session will allow you to better understand the information that you receive (via the media, Internet ...)? | Not at all | **Ο Ο Ο Ο Ο** | Completely | 4.6/5 |
| Do you think this training session will allow you to better discuss with doctors and the medical staff? | Not at all | **Ο Ο Ο Ο Ο** | Completely | 4.4/5 |
| Do you have a more precise idea of the work of a researcher? | Not at all | **Ο Ο Ο Ο Ο** | Completely | 4.7/5 |
| Would you like to follow this kind of training sessions again? | Not at all | **Ο Ο Ο Ο Ο** | Completely | 4.9/5 |
| Would you recommend this training session to others? | Not at all | **Ο Ο Ο Ο Ο** | Completely | 5.0/5 |
