# Supplementary material for: An Innovative Lab-Based Training Program to Help Patient Groups Understand Their Disease and the Research Process
Source: PLoS Biol. 2015 Feb 10;13(2):e1002067. doi: 10.1371/journal.pbio.1002067 (PMC4323103; doi:10.1371/journal.pbio.1002067)
Supplement: S3 Table — (DOC) [file pbio.1002067.s003.doc]

**Supporting Table S3: other indicators of success of the training programme**

| **Type of indicator** | **Partner involved** | **Details** |
| --- | --- | --- |
| National Award | Roche Foundation for chronic diseases | 2010 Award for innovation (includes €30,000 funding) |
| Demand from national organizations for tailored training programmes for patient groups | Inserm (French National Institute for Medical Research) | Organization of one-day seminars for patient groups on general themes: genetic testing, auto-immunity, and inflammation (230 associations, 2005-2013) |
|  | \| Association Francois Aupetit (Crohn’s disease and Chronic ulcerative colitis) \| \| --- \| | Organization of two practical training sessions on biotherapies |
| Demand for scientific consultancy | | Association Francois Aupetit (Crohn’s disease and Chronic ulcerative colitis) | | --- | | Scientific consultancy for a film^(*)^ |
| Demand for a transfer of skills from national and international organizations | French-speaking federation of DNA schools | In 2007 |
|  | Institute of genetic pathology, (Charleroi, Belgium) | In 2010 |
|  | Descartes University (Paris, France) | In progress |

(*) “Biotherapies … patients talk about these treatments”, 2014

(**) DNA schools of Angers, Evry-Génethon, Nîmes, Poitiers
