# Supplementary material for: An Innovative Lab-Based Training Program to Help Patient Groups Understand Their Disease and the Research Process
Source: PLoS Biol. 2015 Feb 10;13(2):e1002067. doi: 10.1371/journal.pbio.1002067 (PMC4323103; doi:10.1371/journal.pbio.1002067)
Supplement: S1 File — (DOC) [file pbio.1002067.s004.doc]

**Supporting File S1: Testimonies from trainees and researchers** (translated from French)

**Testimonies from trainees**

“Back to school at this age, is that sensible? The answer is yes, we have spent three terrific days: studious but relaxed, informative but fun. We left the course, perhaps not more intelligent, but certainly better informed and with our minds more open to the world of re­search. We recommend this training to whoever wants to be educated and come to understanding. Looking forward to return…”

Roger (Huntington disease)

“For the first time, I have enjoyed going to school! And now I itch to go back! I have truly appreciated your consi­deration. I left with plenty of new ideas and of hope. Thank you for your sympathy and warm welcome. I had a wonderful time and learnt a great deal.”

Raphaël (Renal genetic disease)

“By using clear theoretical presentations and hands-on expe­riments, the introduction to molecular biology and bioche­mistry in Marseille has enabled us to understand these techniques, which seemed so complex that we hesitated to enrol in the training. The training session has successfully brought these concepts within our reach, and revealed a glimpse into knowledge that will help us to understand the mechanisms that cause our pathologies.”

Auguste (FSH disease)

« Thanks for the training sessions which allow patients to really take ownership of our disease and to better understand how it works! I stay convinced that to better understand, and thus to better accept, is already a big step towards improving our quality of life. To better know our enemy is to demystify it and to find the means to fight it. I wish you continued success, and thank for all the help you provide against rare diseases and thus to patients... who are not so rare! »

Valérie (Behçet disease)

**Testimonies from researchers**

“These training sessions give us a direct access to associations of patient representatives outside of the “conventional” frame of the doctor/patient relationship. The sessions gave me the opportunity to find the words and phrasings that worked best to explain a particular point (...). I was also able to identify some new aspects relevant to patient management. As a result, we integrated some themes that are important for patients into our therapeutic educational programs, or even in consultation.

Dr Laurent Chiche, MD (Aix-Marseille University)

“I had never become aware of the war that families (of patients afflicted with cystic fibrosis) wage against the opportunistic bacterium Pseudomonas Aeruginosa. I left more convinced than ever of the usefulness and necessity of finding alternatives to antibiotics to fight this pathogen. This is very gratifying for our team, despite the fundamental nature of our work.”

Dr Sophie Bleve (Aix-Marseille University)

« The feedback I got after the training sessions was really positive. I want to congratulate you for the quality of your organization and for the pedagogical concept. Thanks! »

Professor A. Veyradier, MD (Clamart Hospital).

« I very much enjoyed meeting patients and their relatives. These are privileged moments. Thanks and congratulations for what you do. I look forward to hearing about your new project of a training programme on clinical trials, in which I am very interested »
